# The Transactions of the New York Academy of Medicine

**Published:** 1851-10

**Authors:** 


*The Transactions of the New York Academy of Medicine.* Instituted 1847. Vol. I., Part I. Printed for the Academy. New York: Published by S. S. and W. Wood, 261 Pearl-st. 1851. 8vo. pp. 165.

This is the first publication issued under the sanction of the New York Academy of Medicine. It is to be presumed, although not so announced, that it is the beginning of a series which may be expected to follow. The present publication contains eleven papers, most of which are of a practical character, contributed by distinguished members of the profession, and they will be read with pleasure and profit. We hope the Academy will be encouraged by the general interest manifested toward this publication, to carry out the plan of issuing their Transactions at stated times. We append the titles of the several papers, with the names of the respective authors:

Historical Sketch of the Institution for the Insane in the United States of America. By Pliny Earle, M. D.

Report of a Committee appointed by the Academy of Medicine upon the Comparative Value of Milk formed from the slop of Distilleries and other food. By Augustus K. Gardner, M. D.

On the Diagnosis of Yellow and Bilious Fevers. By Ashbel Smith, of Texas, M. A.

Essay on the Value of the Seton, as a remedy in Ununited Fractures; illustrated by Cases. By Valentine Mott, M. D.

Remarks on the Importance of Anæsthesia from Chloroform in Surgical Operations; illustrated by two Cases. By Valentine Mott, M. D.
Of Laceration of the Corpus Cavernosum Penis, commonly called Fracture of the Penis; illustrated by two Cases. By Valentine Mott, M. D.
Cases of Tracheotomy, with Observations. By W. H. Van Buren, M. D.
A Case of Croup in which Tracheotomy was successfully employed. By Gurdon Buck, Jr., M. D.
Amputation of the Thigh, and subsequent Amputation at the Hip Joint, followed by perfect Recovery. By W. H. Van Buren, M. D.
On the Purity and Use of Chloroform. By J. T. Metcalfe, M. D.
A Case of Aneurism, and Ligature of the Left Subclavian Artery, attended with peculiar circumstances. By Valentine Mott, M. D.

